# Variation in zoobenthic blue carbon in the Arctic's Barents Sea shelf sediments

**DOI:** 10.1098/rsta.2019.0362

**Published:** 2020-08-31

**Authors:** T. A. Souster, D. K. A. Barnes, J. Hopkins

**Affiliations:** 1 Ulster University, Coleraine Campus, Coleraine, UK; 2 Biological Sciences, British Antarctic Survey, UKRI, Cambridge, UK; 3 Marine Physics and Ocean Climate, National Oceanography Centre, Liverpool, UK

**Keywords:** zoobenthic, blue carbon, benthos, Arctic, Antarctic, sea ice

## Abstract

The flow of carbon from atmosphere to sediment fauna and sediments reduces atmospheric CO_2_, which in turn reduces warming. Here, during the Changing Arctic Ocean Seafloor programme, we use comparable methods to those used in the Antarctic (vertical, calibrated camera drops and trawl-collected specimens) to calculate the standing stock of zoobenthic carbon throughout the Barents Sea. The highest numbers of morphotypes, functional groups and individuals were found in the northernmost sites (80–81.3° N, 29–30° E). Ordination (non-metric multidimensional scaling) suggested a cline of faunal transition from south to north. The functional group dominance differed across all six sites, despite all being apparently similar muds. Of the environmental variables we measured, only water current speed could significantly explain any of our spatial carbon differences. We found no obvious relationship with sea ice loss and thus no evidence of Arctic blue carbon–climate feedback. Blue carbon in the Barents Sea can be comparable with the highest levels in Antarctic shelf sediments.

This article is part of the theme issue ‘The changing Arctic Ocean: consequences for biological communities, biogeochemical processes and ecosystem functioning'.

## 1. Introduction

Arguably the most visible physical response to global climate change has been ice and snow losses from high latitudes and altitudes. This has been particularly pronounced in the ocean, mainly throughout the Arctic and West Antarctica. The loss has manifested mainly as reductions in maximal seasonal sea ice extent in time and space over continental shelves [1], but until 2014 there were marine ice gains in parts of East Antarctica [2]. Marine ice losses such as seasonal sea ice, ice shelf disintegration and glacier retreat have also contributed to more polar open water [1–3]. These in turn are driving some considerable and diverse knock-on biological responses at high latitudes [4,5]. The effects of ice losses, warming and acidification on the carbon cycle, storage and sequestration are key issues because of potential for feedbacks on climate change. The Arctic carbon cycle is thought to be sensitive to aspects of climate change [6], particularly in terms of storage magnitude and vulnerability of (carbon within) organic matter and methane, in terms of both storage and flux. New major multidisciplinary research initiatives, such as the Changing Arctic Ocean programme (https://www.changing-arctic-ocean.ac.uk/), are underway to try to better understand and predict current uncertainties in marine-based carbon flux.

On land, negative (mitigating) feedbacks on climate from retreating snow and ice coverage have been smaller than expected [7]. By contrast, climate feedbacks on marine continental shelves (from increased storage of carbon by marine life—so-called blue carbon) around West Antarctic seas [8] have been found to be surprisingly large, though complex. Such work has found that the biggest zoobenthic growth increases (and therefore also of blue carbon storage) inshore are short-lived because they are wiped out by coincident increases in iceberg scouring. However, in deeper water marine ice losses are leading to major gains in stored (and probably sequestered) carbon, at least around parts of Antarctica [8–10]. Reductions in annual sea ice losses have been much greater in time and space in the Arctic, but as in West Antarctica are likely to prove complex in terms of blue carbon change. Arctic open water extent correlates with stimulation and maintenance of phytoplankton blooms [11]; however, seasonal sea ice also supports substantial under-ice algal blooms [12]. Arctic and sub-Arctic pelagic biota clearly have a considerable role in carbon storage [13], yet how much of that carbon escapes recycling through the water column and then benthic microbial loops remains to be determined. The Barents Sea has been very well sampled in terms of benthos, even in just the last decade. Cochrane *et al.* [14] and Michalsen *et al.* [15] demonstrated the variability in numbers and abundance of species. Across the Arctic, Roy *et al.* [16] and Jørgensen *et al.* [17] both undertook extensive trawl programmes to record variability in composition, abundance and wet mass. For faunal percentage cover, relative abundance, biodiversity and species monitoring (e.g. with respect to climate) the literature to date is robust. However, most prior studies use non-quantitative apparatus such as Agassiz trawls and unreliable indicators of carbon stock, typically wet mass. This means very little if any literature can be converted to blue carbon per unit area and thus comparison with blue carbon elsewhere. Thus zoobenthic mass still remains little considered in terms of blue carbon storage (but see [18]), yet benthos there can attain great age [19], storing carbon for residence times well inside United Nations (UN) definitions of sequestration (https://static1.squarespace.com/static/54ff9c5ce4b0a53decccfb4c/t/59244eed17bffc0ac256cf16/1495551740633/CarbonPricing_Final_May29.pdf).

This study aimed to establish zoobenthic ‘blue carbon' stock and its variability in space throughout the trough habitat of the Barents Sea. This is an obvious, necessary step prior to investigating potential change in Arctic benthic blue carbon stock or accumulation rate. It was important that any method established during the Changing Arctic Ocean Seafloor (ChAOS) project incorporated a representative range of conditions and measurable environmental factors but also was directly comparable with wider datasets, such as from West Antarctic and sub-Antarctic continental shelves [10]. The Barents Sea is ideal for this as it spans about 12° of latitude, is undergoing historic losses in seasonal sea ice, is where Atlantic and Arctic water masses meet and has a wide range of different substratum and basin versus mid-shelf trough environments. Our key questions were as follows. (i) How much zoobenthic carbon is held per unit area on the shelf north of the Atlantic? (ii) How much does this vary in space and what drives this? (iii) Finally, how do stock levels compare with those in West Antarctica?

## 2. Methods

**Figure 1.**
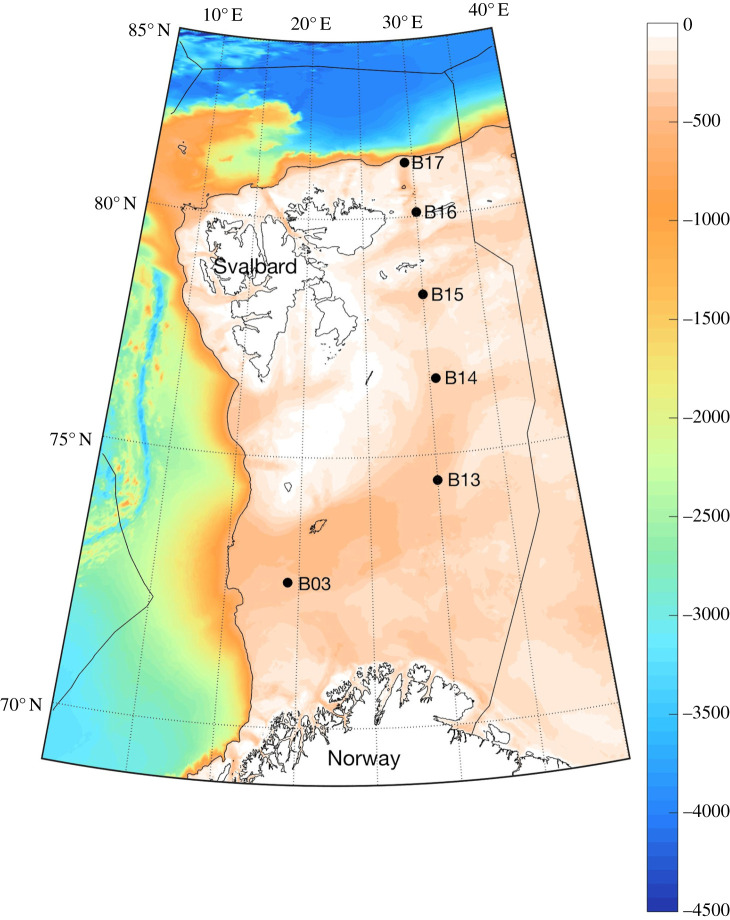
Locations and depth range of the ChAOS field sites in the Barents Sea which were used in this study. (Online version in colour.)

Six sites, from 10 km to 100 km apart, were selected throughout the Barents Sea incorporating a wide range of conditions from the southernmost shelf edge to the northernmost shelf break (figure 1). Within each of these sites we further selected three or more locations kilometres apart to provide nested samples. Sampling took place in the summer of 2017 during the scientific cruise JR16006. A vertical, calibrated, high-resolution camera tripod (Shelf Underwater Camera system (SUCS)) with twin 2000 lumen dimmable lights and an ultra-short baseline positioning beacon was used to take images of the seafloor. To minimize distortion and error, the camera was always perpendicular to the seabed (as opposed to being vertical in the water column), with a mid-focal length (F11) and flat glass port, such that error within the field of view was ± less than 1 mm. Finally an Agassiz trawl was used to collect zoobenthos from each site, by means of three replicate 5 min trawls. Using the SUCS, we captured 586 high-resolution (12 MB) images, each of which was 406 × 341 mm; these were then analysed for the density of each of 14 functional groups. These functional groups were pioneer sessile suspension feeders, climax sessile suspension feeders, sedentary suspension feeders, mobile suspension feeders, deposit-feeding crawlers, deposit-feeding vermiform, deposit-shelled infauna, grazers, soft-bodied sessile predators, calcareous sessile predators, soft-bodied mobile predators, calcareous mobile predators, arthropod predators and flexible (mixed strategy), as used in Antarctic and sub-Antarctic datasets [20]. Functional groups were assigned on the basis of the literature (e.g. [20]) applied to identified morphotypes in images and closer inspection of trawl specimens (matched to similar morphotypes in images). Antarctic data (collected in the same way) have verified that functional groups work as effective surrogates (which therefore lose little information in comparison) for species identity-level data. Trawled sample specimens were separated into morphotypes and functional groups from which morphometrics were measured. Each morphotype was then dried at 60°C for 24 h and weighed. The individual morphotypes were then ashed at 475°C for 12–24 h and reweighed. Approximate carbon content was calculated as 50% of organic mass (dry mass – ash-free dry mass; see [21,22]) plus 12% of ash (skeleton) mass (approximate proportion of carbon in carbonate by molecular mass).

Near-seabed (typically within 10–15 m of the seafloor) measurements of temperature, salinity, chlorophyll-*a* fluorescence and oxygen were obtained from CTD (conductivity–temperature–depth) casts at each site using an SBE 911plus fitted with an auxiliary SBE43 oxygen sensor and a Chelsea MKIII Aquatracka fluorometer [23]. Salinity and oxygen were both calibrated against *in situ* samples. A 75 kHz RD Ocean Surveyor Acoustic Doppler Current Profiler fitted to the ship's hull provided profiles of horizontal current velocities, from 20 m below the sea surface to 20 m above the seafloor [24]. Raw data were collected in 8 m depth bins every 1 s using a bottom-tracking configuration. Single-ping, bin-mapped, Earth-coordinate data contained within the proprietary VMDAS binary files were read into Matlab for further processing and quality control. The single-ping acoustic Doppler current profiler (ADCP) time stamps and the position, attitude, time and heading information from the ship's Seapath320+ were also ingested into Matlab. Ensembles with no ADCP data or missing heading information were removed. The single-ping ADCP velocities were rotated from the vessel's centreline to a True North reference and transducer misalignment errors were corrected for. Further screening was performed to remove data where there was a low signal-to-noise ratio in any bin; where a four-beam solution was not possible; where the maximum change in heading between pings was greater than 10° per ping; where the maximum change in the ship's velocity between pings was greater than 0.55 m s^−1^ per ping; and where the error velocity was more than twice the standard deviation of error velocities of a single-ping profile. Velocities close to the bottom that were contaminated by strong reflections from the seafloor were also discarded. Lastly, 5 min averages were created and the absolute water velocities were determined. Depth mean velocities comprising the barotropic tidal velocity and any non-tidal contribution to the depth average flow (e.g. a density-driven geostrophic current) were calculated. The ship worked at each site for 30–42 h; therefore, between 2.5 and 3.5 semi-diurnal tidal excursions were resolved.

Each site was sampled during a different phase of the spring–neap tidal cycle. To ensure that any potential relationship between zoobenthic carbon and velocities was not masked (or indeed artificially generated) by the spring–neap range in current speed at each location, we generated tidal current predictions using the Oregon State University (OSU) Tidal Inversion Software (OTIS) [25] and the regional Arctic 5 km solution. The tidal prediction at each site was based on eight harmonic constituents (including M_2_, S_2_, N_2_ and K_2_). We extracted the maximum tidal current velocities on 19 and 26 July 2017, the nearest neap and spring tides to our sampling times, respectively.

Substratum type (Wentworth scale) and rugosity (shadow length) were estimated from SUCS images. Substratum type was graded to categories from clay to bedrock. Shadow length in images was used to grade rugosity (seabed roughness) into five categories from smooth (1 mm level roughness) to rough (40+ cm level differences). Sea ice history was calculated as the number of sea ice days per year across 16 years (in which a sea ice day was classified as having over 85% sea ice concentration) using the 6.25 km data, AMSRE for January 2011–October 2011, SSMI for November 2011–July 2012 and AMSR2 for July 2012–December 2018 [26].

The number of individuals within each of the 14 functional groups was converted into densities per area (m^2^). For each image, the mean carbon mass of each functional group (per site) was multiplied by the total number of individuals from this functional group and summed to give a standing stock of epi-zoobenthic carbon per image/area. Thus, for each site, there were approximately *n* = 20 values of blue carbon stock (20 images were taken at each deployment). For comparison with West Antarctic data, Arctic sites were categorized into shallows, rocky rubble, sediment basin or sediment shelf break types.

Differences in data were tested where possible using ANOVA; where this failed to meet assumptions of normality or equality of variances differences in data were transformed (log). Where data failed to comply with equality of variance but did comply with normality, we used Welch's one-way ANOVA. Arctic versus Antarctic data still showed significant non-normality and inequality of variance after BoxCox transformation; however, ANOVA was considered to be robust enough to use with the transformed data. Multivariate analyses, using routines within the PRIMER statistical package (PRIMER-e Ltd, Plymouth, UK), were used with assemblage data, which were summed into areas totalling 0.25, 0.5 and 1 m^2^. This summing was undertaken by pooling the fauna from two randomized images (from the same deployment for 0.25 m^2^, pooling four randomized images for 0.5 m^2^ and eight images for 1 m^2^) and similarly investigated using non-metric multidimensional scaling (nMDS), using both morphotypes and functional groups for comparison. Blue carbon mass data were also compared using nMDS, principal component analysis (PCA) and the BEST (BIO-Env) procedure to correlate biological patterns with environmental data (latitude, depth, temperature, oxygen, chlorophyll-*a* fluorescence, salinity and substratum). Current flow data were collected per site not per sample so were not included in the analysis. The BEST procedure was employed to find the best match between the assemblage data and those from environmental variables associated with these samples, namely the best explanatory variable, and then RELATE to statistically analyse the BEST procedure to see how related the two matrices (blue carbon data and environmental variables) are; if there is no relation, *ρ* will be approximately zero.

## 3. Results

### (a) Environmental differences between sites

For many explanatory variables, there was little difference among sites, despite them being considerable distances apart (table 1). For example, variability in salinity and oxygen concentration among all sites was biologically minor (e.g. 34 practical salinity units (PSU) and 46 μmol kg^−1^, respectively).

**Table 1.** Environmental characteristics of Barents Sea sites in July 2017. Sea ice is given as mean number of days per year since 2002. Maximum depth average velocities in m s^−1^ recorded during the sampling of each site and the maximum predicted spring tide velocity (m s^−1^) on 26 July 2017. Near-bed temperature in °C. Salinity is shown in PSU, oxygen in µmol kg^−1^ and chlorophyll*-a* in µg^−1^. Substratum is grain size and rugosity is the scale of variability in roughness.

	depth	sea ice	max. observed flow	max. predicted tidal velocity on 26 July 2017	temp.	salinity	oxygen	chloro.	substratum	rugosity
B3	367	0	0.15	0.21	3.95	35.04	274.0	0.02	silt and rock	cm
B13	358	0	0.12	0.14	1.78	35.00	294.7	0.06	silt	mm
B14	293	6.2	0.1	0.11	1.95	34.99	276.6	0.04	silt	mm
B15	316	42.8	0.1	0.08	−1.49	34.89	314.9	0.03	silt	mm
B16	310	45.9	0.25	0.25	−1.44	34.67	320.0	0.02	silt	mm
B17	314	50.4	0.4	0.21	1.76	34.89	293.4	0.02	silt and rock	cm

Changes in the near-bed temperature and salinity between the sites were consistent with the transition from warm (greater than 3°C) saline inflow of Atlantic water across the Barents Sea Opening at B3 to cold and fresh Arctic water northeast of Svalbard. Sites B13 and B14 were both 2°C colder than B3. The temperature of bottom water at B15 and B16 was sub-zero. Despite being further north, B17 was 3°C warmer than B15 and B16. Its near-shelf break location means that B17 was probably influenced by warm Atlantic-origin waters being carried in the Arctic boundary current.

The maximum observed velocities and predicted spring tidal current are summarized in table 1. The maximum observed velocities recorded at each site ranged from 0.1 m s^−1^ (at B14 and B15) to 0.4 m s^−1^ (at B17). Note, however, that sites B15 and B17 were sampled during neap and spring tides, respectively, which, based on only 2 days of data, is likely to amplify the apparent difference between them. Although the current velocities from the tidal prediction model are typically biased towards lower velocities (and do not account for any non-tidal processes), a comparison of the maximum predicted spring currents at each location helps to even out this potential aliasing across all sites. The strongest tidal currents (0.21–0.25 m s^−1^ on 26 July 2016) were predicted to occur at B3, B16 and B17. The difference between the predicted maximum neap and spring tide currents at each site ranged from 0.05 m s^−1^ at B15 to 0.16 m s^−1^ at B16. This demonstrates that the temporal variability in current magnitude at each site is almost as high as the inter-site differences. At sites B13 and B17, there was a northeast depth mean non-tidal flow of 0.03 m s^−1^ and 0.07 m s^−1^, respectively. Overall, we conclude that sites B3, B16 and B17 experience the largest currents. Sites B13–B15 typically experience weaker currents.

The sites B3 and B17 had occasional boulders, and so raised rugosity but only in their immediate vicinity. However, the combination of high flow and boulders at B17 is potentially important for the establishment of suspension feeders.

**Figure 2.**
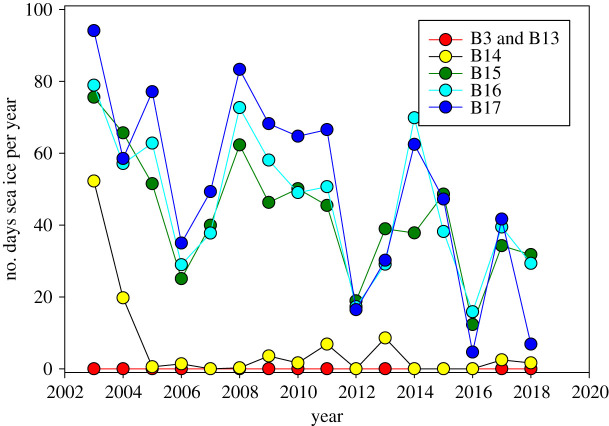
Number of sea ice days per year at each location between 2002 and 2018, with B3 being Atlantic and open water all year round. (Online version in colour.)

The number of sea ice days per year at each site was more predictably aligned with latitude and the polar front position. The three southernmost sites (B3, B13, B14) had little or no seasonal sea ice cover. The three northernmost sites (B15–B17), however, have been experiencing a significant reduction in the number of sea ice days per year over the last decade, from 80 to 90 days in 2002 to less than 20 in 2016 (figure 2).

### (b) Functional groups and surrogacy

**Figure 3.**
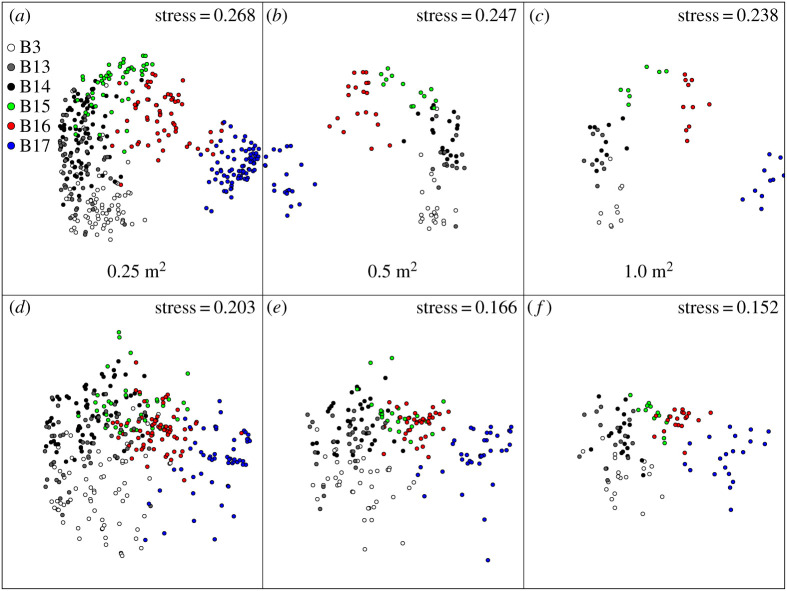
nMDS ordinations of taxonomic and functional group assemblages across three spatial scales (0.25, 0.5 and 1 m^−2^) at all six locations. Taxonomic data are the number of morphotypes per sample at (*a*) 0.25, (*b*) 0.5 and (*c*) 1 m^2^ and functional group refers to the numbers of functional groups per sample at (*d*) 0.25, (*e*) 0.5 and (*f*) 1 m^2^. (Online version in colour.)

**Figure 4.**
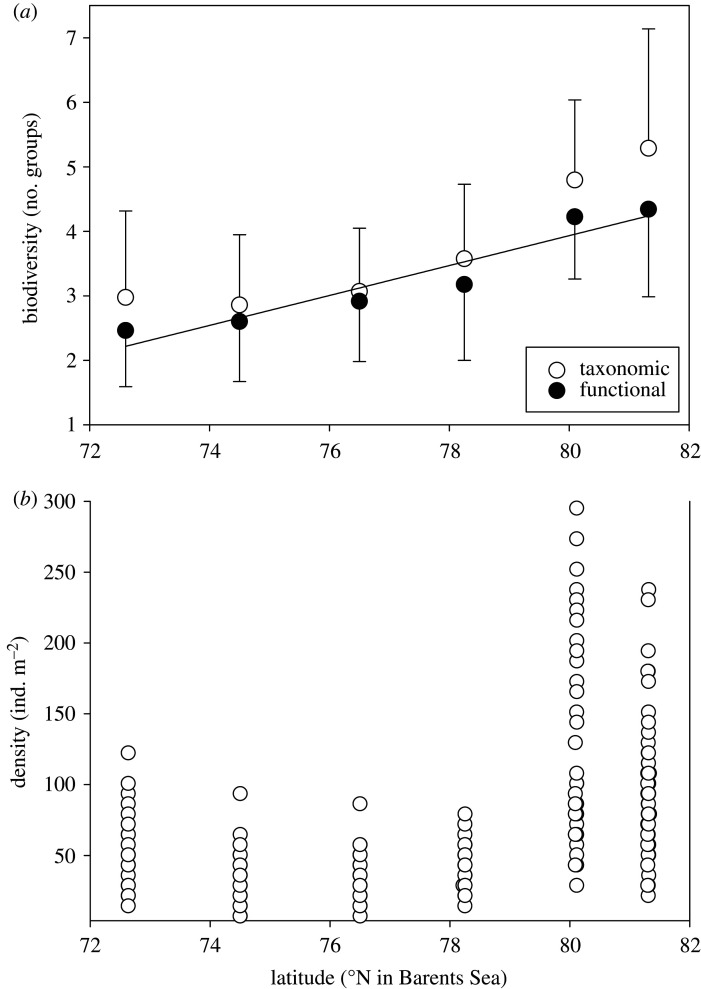
Number of taxonomic and functional groups (*a*) and density of benthic marine invertebrates (*b*) at each of the six locations within the Barents Sea in increasing latitude. Richness data (*a*) are the mean and standard deviation (bars) of numbers of morphotypes (termed taxonomic) and numbers of functional groups (termed functional). Density (of individual macrofauna) data (*b*) are shown per sample.

Ordinations of assemblage composition of 0.25, 0.5 and 1 m^2^ areas using separate taxonomic and functional categories showed similar patterns. There was more dispersion in functional group plots of the same area size, but the nature of the trend (a clear latitudinal cline from B3 to B17 site) was similarly evident. Site B13 was least distinct (in both taxonomic and functional ordinations) owing to the overlap with B3 and B14, the sites immediately south and north of it, respectively. Thus functional groups showed good taxon surrogacy with compositional similarity within and between sites (figure 3). While taxonomic richness showed no simple pattern with latitude (although the richest were the two highest latitude sites, B16 and B17), functional groups showed an increase northwards (figure 4; ANOVA, *F* = 191.6, *p* < 0.001). The range of functional richness was one to eight functional groups per sample (image), while the taxonomic richness ranged from one to 12 morphotypes per sample. As with richness, both magnitude and variability in density were highest above 80° N, but all other sites were little different (figure 4*b*). Establishing these patterns and variability in zoobenthos is important to set the context of blue carbon findings.

**Figure 5.**
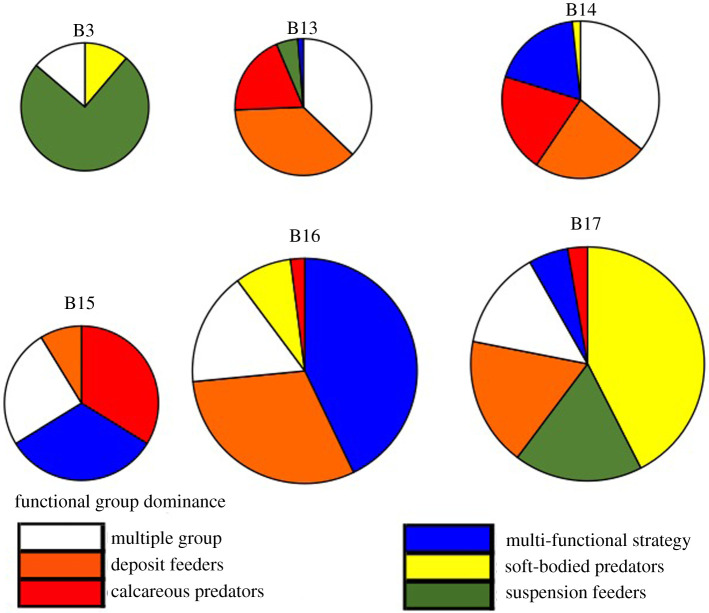
Different functional groups dominating across the six seabed field sites, where pie size is representative of the mean number of the functional groups at that location. The smallest pie contains three dominant functional groups and the largest six. (Online version in colour.)

The most abundant functional group differed within (replicates) and between sites, such that the patterns of dominance were ‘site specific' (figure 5). Suspension feeders comprised most of the fauna at B3 but were virtually absent at B14 and B15. There were no clear environmental signals to such dominance. For example, sites with lower sea temperature were no more similar with respect to any one group's dominance than sites with increased temperatures. Multi-feeding trait groups, such as ophiuroids, mainly dominated at sites with sea ice, but seemingly not in any proportion to sea ice probability or duration. Suspension feeders were most dominant in the two shelf-edge sites (B3 and B17) and calcareous predators were more dominant at sites with lower current speeds, but in each case these were highly variable within sites as well.

### (c) Zoobenthic carbon stock

**Figure 6.**
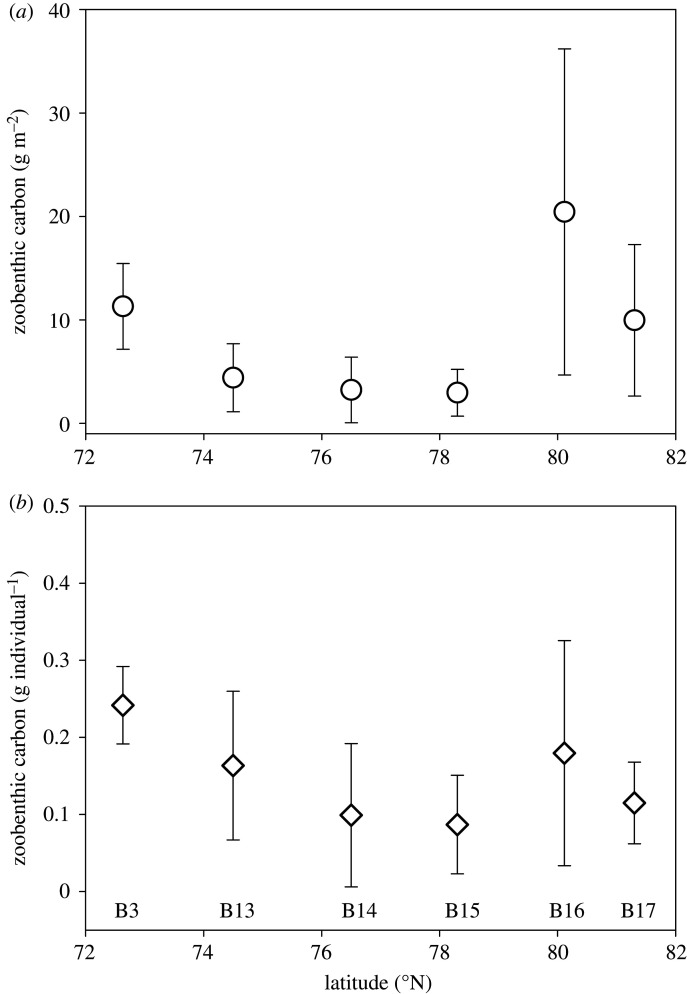
Mean amount of zoobenthic blue carbon (g m^−2^, *a*) and zoobenthic carbon per individual species (g individual^−1^, *b*) at each location with increasing latitude, with standard deviation (bars).

Mean zoobenthic carbon varied from less than 3 g m^−2^ to greater than 20 g m^−2^ and showed a similar pattern across sites for zoobenthic density (figure 6). There was a significant difference in zoobenthic carbon across sites (one-way ANOVA, *F* = 105, *p* < 0.001) with post hoc Tukey tests showing significantly more seabed carbon at B16 than any other site. However, there were no significant differences in carbon per zoobenthic individual (figure 6*b*), so the higher carbon standing stock was due to densities (figure 4*b*) and the functional group composition (figure 5). At B16, an unusually important functional group was deposit feeders (figure 5). These included many polychaetes, bivalve molluscs and echiurans, which were larger than at other sites (one-way ANOVA, *F* = 89, *p* < 0.01; B16 0.45 g per individual versus other sites mean 0.16 g per individual).

**Figure 7.**
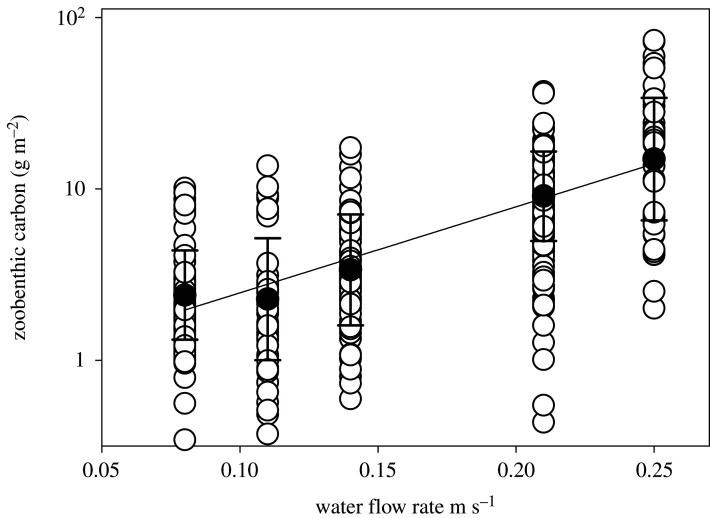
Zoobenthic carbon (g m^−2^) against maximum spring tidal current velocities (m s^−1^) predicted for 26 July 2016 at each site. Open circles are raw data; filled circles are the mean; and the bars are standard deviation.

**Figure 8.**
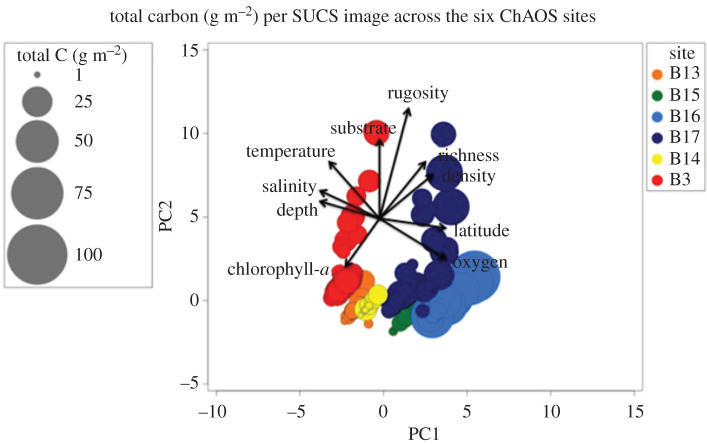
Two-dimensional PCA ordination bubbleplot of total carbon (g m^−2^) at the six Arctic sites in the Barents Sea (stress = 0.01, square root transformed and normalized). (Online version in colour.)

From the environmental factors we considered, obvious drivers of variability in functional group importance did not emerge in our data. For example, high-carbon sites (figure 6) were associated with high and low sea ice, sea temperature, oxygen and salinity. At site level, the highest zoobenthic carbon was found at high-velocity locations (B3, B16, B17) (table 1 and figure 7). Of the variables we measured, only the current magnitude could significantly explain any of our spatial carbon differences. Zoobenthic carbon showed a significant relationship with the log-predicted spring tidal current speed (one-way ANOVA, *F* = 82, *p* = 0.001) and the *r*^2^ value of the associated regression explained 44% of data variability. Zoobenthic carbon showed distinct clustering for each site (figure 8); however, there was no correlation between total carbon and any of the environmental variables (BEST, BVSTEP, Spearman *ρ* = 0.224, *p* < 1%). The RELATE model also showed little to no correlation (Spearman) between the biological resemblance matrix and the environmental matrix, confirming no obvious environmental drivers behind the zoobenthic carbon stock.

**Figure 9.**
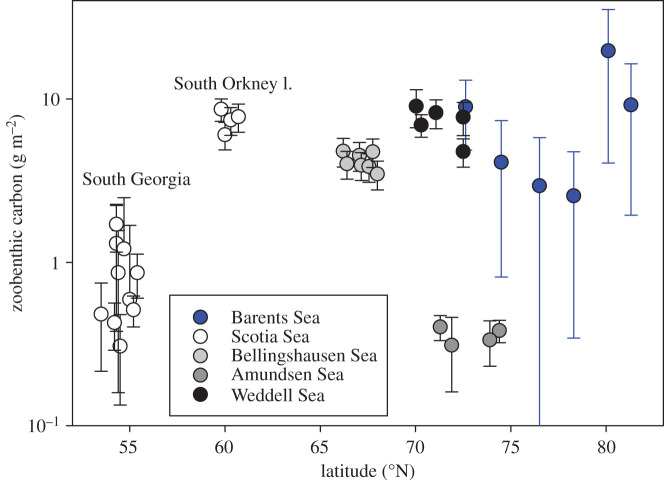
Amount of zoobenthic carbon by latitude in the Arctic, the Western Antarctic and sub-Antarctic. Data are shown as the mean per site with standard deviation. (Online version in colour.)

Comparison of BoxCox-transformed Barents Sea zoobenthic carbon stock data with similarly transformed and comparable Antarctic literature data (figure 9, [8] and electronic supplementary material) found a significant difference between polar regions, among sites and a significant pole×site interaction term (table 2). Both West Antarctica and the Barents Sea have high-carbon areas (particularly South Orkney Islands, Weddell Sea and B16, respectively) and low-carbon areas (South Georgia, Amundsen Sea and B14 and B15, respectively).

**Table 2.** General linear model ANOVA of polar region, site and estimated zoobenthic carbon (BoxCox transformed data) with a pole × site interaction term.

	d.f.	sum sq	mean sq	*F*-value	*p*-value
pole	1	151.9	151.9	179.8	0.001
site	5	106.7	21.3	21.3	0.001
pole × site	4	102	25.5	30.2	0.001
error	805	680	0.8		
total	815				

## 4. Discussion

Antarctic sea ice losses may have led to greater carbon drawdown and seabed storage, much of which probably escapes the carbon cycle through burial [8]. But is this happening in the Arctic? Our Barents Sea data were collected during a very limited time frame but did span a wide range of sea ice durations and yet showed no evidence for influences on benthic blue carbon. Highest blue carbon stocks occurred in the highest and lowest sea ice sites (figures 2 and 6). This is important because until 2014 Arctic sea ice losses dwarfed those in Antarctica. Hence, the response of Arctic oceans is likely to have a big impact on the strength of climate feedbacks on ongoing climate change. The minimum extent of sea ice in the Arctic is currently declining by an average of 91 600 km^2^ per year [27]. Increased negative feedback (mitigation) requires more carbon to leave the carbon cycle or even more carbon drawdown or cycling. Much environmental focus has been on how pelagic primary [11,12] and secondary [13] production changes and how sensitive these changes are to intensifying warming [6]. As carbon sequestration is defined (by the UN) as removal from the carbon cycle for at least 100 years there is very little—if any—scope for this in the water column (few Arctic pelagic biota are thought to exceed 100 years in age). Thus, sequestration requires burial, which can only happen at the sediment–water interface, so the fate of carbon at the seafloor is a crucial part of the cascade regardless of carbon pelagic or benthic origin.

The Barents Sea can be a very productive system, but, as elsewhere, little of this may reach the seabed; for example, only 17–52% particulate organic carbon even reaches 90 m depth at most sites [28] and just 4.4% by 500 m [18]. Seabed carbon from secondary production in the current study in the Barents Sea reflected the biological variability reported from Arctic seabeds [14–18]. Biodiversity [14–18], biomass [14,15,29–31] and thus natural capital of blue carbon standing stock vary massively in the Arctic. Many of the biodiversity and biomass values reported and, by proxy, benthic carbon values are quite low [18,29–30]. However, many such carbon values were derived by conversion of wet mass with Rowe's [32] conversion factor (1 mg C  =  0.034 mg wet mass), which can differ by an order of magnitude between taxa [18] so may have error levels comparable to site differences. However, key baselines in the old and fairly comprehensive literature [33] use such values and methods, so it will remain important to find ways to compare these with modern blue carbon work. The 4–20 g m^−2^ of zoobenthic ‘blue' carbon found in the Barents Sea is higher than for most reported Arctic benthic values but generally falls within the range of literature estimates [29–31,34]. At similar latitudes on the more Atlantic-influenced west coast of Spitsbergen, Gorska *et al.* [31] found a comparable mass of zoobenthic carbon to our values on the shelf, declining by an order of magnitude down the continental slope. They [31] also found that benthic biomass was highest in the Fram Strait, moderate in the North Atlantic and lowest in the ‘Arctic’, further suggesting that these were limited by organic carbon (phytoplankton) flux. Notably one of the highest zoobenthic carbon values found previously in the Barents Sea [29] was near one of our sites, B17. Indeed the zoobenthic blue carbon levels in the Barents Sea fall at the upper end of all polar values (figure 9). South of the Atlantic Ocean, the South Orkney Islands are considered a benthic blue carbon hotspot [35], but our study using similar methods found that half of the Barents Sea values exceeded those. Such data suggest that the Barents Sea may be important for blue carbon natural capital, yet it is also a region in the midst of profound sea ice losses (figure 2), fishing activity [36] and other stressors [6]. The key step following establishment of extent and variability of blue carbon is to elucidate the drivers behind such values and surprisingly we found no links between blue carbon stock and sea ice cover, unlike in West Antarctic seas [8]. This was all the more surprising as less primary production is thought to reach the seabed at the more northerly (more sea ice covered) sites [28].

Although the changes in bottom water temperature and salinity were consistent with the transition from Atlantic to Arctic waters, changes in zoobenthic carbon could not be statistically correlated with the bottom water hydrography. For example, the polar front, which marks the transition from Atlantic to Arctic water, was located just south of B15 during our cruise. However, the largest range in zoobenthic carbon was found north of the polar front between B15 and B16, sites that had very similar bottom water temperatures and salinities. It was surprising not to find any strong relationships between near-seabed environmental characteristics and zoobenthic blue carbon levels. This may be because (i) our sampling was only during a single month so did not capture the variation in seasonal range of conditions, (ii) we focused within a single habitat (shelf trough) where the levels of environmental variability were subtle, (iii) differences are less amplified in epifauna, or (iv) such factors are not particularly important to standing stock (but may be important for other unmeasured aspects of zoobenthic carbon, such as production).

Tidal currents make an important contribution to the mixing and transport regimes in most coastal and shelf seas. Using data from the same TPXO Inverse Tidal Model that we have used here, Holt *et al.* [37] calculate that the mean M_2_ semi-major axis tidal current speed is 0.29 m s^−1^ for water shallower than 500 m, compared with a global mean of 0.06 m s^−1^. The clearest environmental driver of distribution of zoobenthic carbon identified by us was current speed. Increased amounts of zoobenthic carbon were found at sites that experience higher current velocities (figure 7). Organism design is important in enhancing the hydromechanic conditions necessary for feeding. Best [38] showed that feeding rate is a function of ambient flow speed, which affects filtering efficiency, and this could explain the relationship between flow and zoobenthic carbon as there will be an optimal flow rate for filter feeders to draw down enough water. Kedra *et al.* [39] also found considerable variability in community structure and function in the Svalbard Bank of the Barents Sea, which corresponded to currents and overlying water masses. On the SUCS images, 22% of individuals on soft substrata were suspension feeders (50% primary consumers). Growth of suspension feeders can be enhanced at higher flow speeds because of increased turbulent vertical mixing [40]. Why would other feeding modes be boosted by higher current flow, though? Currents could transport phytoplankton under seasonal ice (from open water) to provide food for suspension and deposit feeders, but also carry zooplankton and detritus for more predatory/scavenging feeding modes. However, the upper limits for feeding are determined by the balance between energy gained from feeding and the cost of filter feeding [41]. There is a cost to feeding (measured as raised metabolic activity (oxygen usage) to support higher functionality) and therefore benthos may choose not to feed even though food is present as the benefit of feeding would depend on both the quality and quantity of the available food [42].

Current speed is important for megafaunal distribution [43], and also physical dynamics can play an important role with fauna shifting to suspension feeders in dynamic areas and deposit feeders in depositional areas [33,34,44]. In this study, B13, which was a site of lower flow rate, had a much larger proportion of deposit feeders than suspension feeders; however, B16, which experiences stronger currents, had both suspension and deposit feeders present.

During the winter, ocean-to-atmosphere heat loss, strong winds and, in polar regions, brine rejection during sea ice formation drive strong vertical mixing of the water column. On continental shelves, where the depth of vertical mixing exceeds the total water column depth, the sea floor and its blue carbon reservoir may become seasonally ‘re-connected' to ocean–atmosphere processes and exchanges and therefore experience seasonal variability in temperature, salinity and oxygen concentration. The impact that seasonal variability of these environmental variables has on benthic blue carbon cannot be determined from this dataset.

Future projections show that increased rates of momentum transfer from the winds into the ocean [45], increases in volume, heat and freshwater transports from the Pacific [46] and warming of Atlantic water entering the Arctic [27,47,48] are changing the hydrography and circulation of the Arctic Ocean. Whether or not any changes in wind-, tidal- or density-driven current regimes across the Arctic shelves will benefit the Arctic benthos or not is unknown.

Lifespan or trophic position of biota need not necessarily impact their blue carbon importance, because the key climate relevance is whether the carbon is sequestered, i.e. through burial. Intuitively it could be argued that higher predators probably bury proportionally less carbon because of losses due to compound inefficiencies and respiration losses up the food chain. A major likely influence on lifespan and likelihood of biomass burial is trawling. The impact of trawling on macro-zoobenthos [36] may hinder the observation of a relationship between sea ice loss and benthic blue carbon increase. Trawling may prevent long-term deposition of carbon by grinding up benthos and reworking the sediment on impact with the seafloor [49], similar to ice scour disturbance in the Antarctic [50]. B13, B14 and B15 were all low in terms of benthic blue carbon stock compared with the other sites; however, these sites are in an area of intense trawling activity [51]. The trawling for fish and shrimp in the Barents Sea is causing a reduction in total benthic biomass [51]. North of our sites in the Arctic Ocean, the area was covered by sea ice for more than 200 days per year [51]. This area is also high in deposit feeders (figure 5) and is protected as it is outside the current range of trawling, but this could change as sea ice declines and exploitation moves northward. Patchiness within and between sites could be due to the local catastrophic trawl events as each location could be at a varying state of recovery [10].

The Arctic and the Western Antarctic are ‘hot spots' for climate change (or sustained periods of warming) [1,2,4,6]. Barnes [52] showed that the loss of sea ice in the Western Antarctic Peninsula increased the duration of phytoplankton blooms, which led to an increase in zoobenthic blue carbon and consequently carbon drawdown and thus provided a negative feedback on climate change. Our study showed that zoobenthic blue carbon in the Barents Sea was considerably higher than that in the sub-Antarctic (South Georgia), and it was twice the amount commonly found in the Antarctic with the exception of shallow Antarctic systems. However, perhaps of more note was the high variability in Barents Sea benthic carbon (figure 9), particularly at site B16 (figure 4) compared with the Antarctic shelves. The reason for this is unclear, although South Georgia also has high variability in benthic carbon stocks and, like the Barents Sea, also has high site-to-site variability in functional group presence and dominance [20]. At South Georgia that was driven by major habitat and substratum differences, which was not the case in the Barents Sea. Overall, our data show that there is a high level of zoobenthic carbon in the Barents Sea section of the Arctic where sea ice levels have been retreating significantly [17]. The polar front may be responsible for a multi-year signal of food supply to the seafloor that could be reflected in benthic communities. Carrol *et al.* [44] found that infaunal organism density and species richness were, respectively, 86% and 44% greater at stations located near the polar front than at stations located in either Atlantic- or Arctic-dominated water masses. This is not clear in our epifaunal data as B16 is in Arctic water and had a higher amount of taxonomic diversity and density than the sites around the polar front; however, this signal may only be picked up in multi-year data. We found 19–36 morphotypes of epifauna per site (at B14 and B17, respectively), which was low to comparable to past regional soft sediment work. Previous richness levels found included 96–179 per site in the Barents Sea [14], 22–85 in the Kara Sea [30] and 10–64 in Canadian Arctic shelf sediments [16]. Meaningful comparisons of such values are very difficult though because of differing sample areas, efforts and equipment types.

In conclusion, our study answered the questions we set out to investigate; we found more functional groups northwards in the Barents Sea with different functional groups dominating and each site being unique. Blue carbon storage showed both ‘within site’ and ‘between site' variation for which trawling could have been a contributing factor. The blue carbon stock of the Arctic Barents Sea was twice that of the Antarctic soft sediment continental shelf and could therefore have great potential for increased carbon drawdown with possible values of around 1–6 t km^-2^ per year. One of the main factors underpinning the distribution of zoobenthic blue carbon appears to be flow rate. Arctic shelves differ substantially from those along the Western Antarctic Peninsula, where most research for the potential for enhanced blue carbon sequestration caused by sea ice loss has been carried out. Our findings reiterate the need for investigations into zoobenthic blue carbon in the Arctic to better inform global estimates of carbon budgets [8]. Benthic blue carbon responses to sea ice loss in the Arctic could represent one of the largest negative feedbacks to climate change; however, our data show no clear link between sea ice and zoobenthic carbon but provide a good baseline of Arctic benthic blue carbon data for building on with further work. We suggest investigation with a wider range of Barents Sea sites to include habitats different from soft sediment continental shelf, analysis of data from 2018 and 2019 for multi-year comparison and ground-truthing our data in a multidisciplinary context by looking at box-core and multi-core results from the same sites. Other studies [53] used environmental DNA to look at diversity and found a higher diversity than with morpho-taxonomy, which when linked to the environmental variables could give a different answer in response to environmental drivers. Studies combining multiple techniques are required to gain a complete picture of the environmental drivers controlling community structure. There is also a need to look at water column nutrient data for each of our sample sites to see if the nutrients required to sustain phytoplankton blooms for longer are available. Prolonged warming and its predicted consequences are likely to continue to affect the structure and functioning of marine benthos in the Arctic.

## Supplementary Material

Supplementary data for ‘Variation in Zoobenthic blue carbon in the Arctic's Barents Sea Shelf Sediments

## Data accessibility

All data are available from the Polar Data Centre or the British Oceanographic Data Centre, NERC, UK, on request.
